# Evaluating the efficacy of an Advanced Care Planning Program for Health Decisions in patients with advanced heart failure: protocol for a Randomized Clinical Trial

**DOI:** 10.1186/s12872-020-01738-0

**Published:** 2020-10-21

**Authors:** Beatriz Sánchez, Carlos Guijarro, María Velasco, María Jesús Vicente, Miguel Galán, Benjamín Herreros

**Affiliations:** 1 Unidad de Medicina Interna, Hospital Universitario Fundación Alcorcón, Calle Budapest 1, 28922 Alcorcón, Madrid Spain; 2 Instituto de Ética Clínica Francisco Vallés, Universidad Europea de Madrid, Madrid, Spain

**Keywords:** Advanced Care Planning, Heart failure, Advanced directives, Shared decision making

## Abstract

**Background:**

An Advanced Care Planning (ACP) program of health decisions is the result of a process of reflection and relationship-building between the patient, their relatives and health professionals. It is based on respect for patients’ autonomy, involving them in making decisions about their disease in a way that is shared between the medical team, the patient and their relatives. Up until now, the efficacy of an ACP has not been measured in the existing literature, and therefore it is unknown if these programs reach their goal. The main objective of our study is to evaluate the efficacy of an ACP program for decision-making in patients with advanced heart failure (HF) in comparison to usual follow up and care. This objective will be evaluated by the Patient Activation Measure test, which measures the participation and self-management of the patient in decision-making. Secondary objectives: to evaluate the effect of the program on quality of life, to know if the patients wishes expressed through the ACP program are fulfilled, to measure the impact of the program on patients’ caregivers, to determine the satisfaction of patients included in the program and to evaluate the effect on quality of death.

**Methods:**

Randomized multicentre clinical trial at four hospitals in Madrid. Once they are included in the study, patients’ allocation to groups (control vs intervention) will be made by alternative sampling. ACP will be applied to the intervention group, whereas in the Control Group usual follow-up will be carried out in HF units. All patients will fulfil questionnaires and tests related to the objectives of the study again after a 12-month follow-up period in order to gauge the effect of ACP in patients with advanced HF.

**Discussion:**

The characteristics of patients with advanced HF make them a model for designing ACP programs, given the high prevalence of this disease, the progressive increase in its incidence and it’s clinical characteristics. Until now, the efficacy of this type of program has not been measured, so this Clinical Trial can provide relevant data for future ACP projects.

*Trial registration* ClinicalTrials.gov Identifier: NCT04424680. Registered 9 June 2020. Retrospectively registered, https://clinicaltrials.gov/ct2/show/NCT04424680?term=NCT04424680&draw=2&rank=1.

## Background

The prevalence and incidence of heart failure (HF) has significantly increased in recent years, in part due to population ageing. It has become the leading cause for admission to Spanish hospitals in patients over 65 years, and currently accounts for 3% of all hospital admissions and 2.5% of the cost of healthcare [1]. While reduction of mortality and morbidity have been the primary objectives in most of HF studies, we must not forget that HF, in addition to increasing mortality, involves a drastic reduction in patients’ quality of life and leads to deterioration of their functional capacity. Taking into account that patients with HF are often elderly, the negative impact of HF on patients’ autonomy in healthcare decisions can be understood.

The Spanish legal framework clearly states both patients and healthcare professionals rights and obligations regarding the clinical relationship. Law 41/2002 regulating patient autonomy was enacted in 2002 [2]. In this Law—as well as in subsequent legal norms—it is specified that patients have the right to state their will in advance through Advance Directives [3, 4]. However, Advance Directives (AD) have little impact on clinical practice, not only in Spain but also in many other countries [5–8]. The lack of impact on clinical practice of AD, as well as the common belief that they only concern patients with terminal illnesses, has led to the conclusion that decision making at the end of life must not depend exclusively on these written forms. These decisions should be centered on communication among doctors, patients and relatives during the end-of-life process [9–11], according to a specific procedure named Advanced Care Planning (ACP) of health decisions. ACP is a structured approach that allows patients, relatives and physicians to discuss end-of-life decisions. ACP enables individuals to define goals and preferences for future medical treatment and care, to discuss these goals and preferences with family and health-care providers, and to record and review these preferences if appropriate [12]. There is evidence that ACP interventions can improve patient-related outcomes such as patient satisfaction with care, quality of communication and shared decision-making [13].

ACP programs for decision-making have been carried out in Spain in diseases like chronic kidney disease, [14, 15], but have not yet been applied to HF. Until now there have not been studies to determine if ACP programs are effective and, consequently, if they meet their objectives. Therefore it is our objective to carry out a Randomized Clinical Trial to evaluate the efficacy an ACP program for health decisions in patients with advanced HF. The aim of this article is to present our study protocol.

## Methods

### Design and study population

Randomized multicentre clinical trial at the Fundación Alcorcón Hospital (Madrid), Ramón y Cajal Hospital (Madrid), Princesa Hospital (Madrid) and San Carlos Clinical Hospital (Madrid) in order to evaluate the efficacy of an ACP program for health decisions in patients with advanced HF. The study will include outpatients with HF according to Framingham diagnostic criteria, in advanced stage of the disease (defined as stage C or D of the ACCF/AHA classification) and with full capacity to decide. Table 1 shows inclusion and exclusion criteria. Patients will be included consecutively and followed for one year.

**Table 1:** Inclusion and exclusion criteria

*Inclusion criteria*
Patients with HF defined by Framingham diagnostic criteria
Stage C or D of the ACCF/AHA classification
Full capacity to decide
Signing of informed consent
*Exclusion criteria*
Cognitive impairment, measured by Mini-Mental Status Examination (< 27)
Presence of another disease other than HF that may severely affect the quality of life: stroke with significant residual deficit, end-stage renal failure, Child C cirrhosis, extreme obesity, haemoglobin < 8 g/dl, advanced peripheral artery disease (stage III–IV), severe thyroid or adrenal disease, neoplastic with estimated survival of less than 2 years
Patients who do not sign the informed consent

### Objectives

The main objective is to evaluate the efficacy of an ACP program for decision-making in patients with advanced HF in comparison to usual follow up and care. This objective will be evaluated by the PAM (Patient Activation Measure) test, which measures the participation and self-management of the patient in decision making. Secondary objectives are: to evaluate the effect of the ACP program on quality of life, to know if the patients wishes expressed through the ACP program are fulfilled, to measure the impact of the ACP program on patients’ caregivers, to determine the satisfaction of patients included in the program and to evaluate the effect on quality of death. Secondary objectives will be assessed through questionnaires specifically developed for each objective.

### Study procedure

Researchers will attend a training course prior to the commencement of the study and the ACP program, with the aim of learning how to carry out the tests employed in the study. The research staff will prospectively carry out data collection. Candidate patients will be informed orally and in writing about the study prior to signing the informed consent. An informed consent form will be needed to participate in the study. Before being accepted, patients will be required to perform a Mini-Mental Status Test. Those scoring under 27 will be excluded.

Once patients have been included in the study, the assignment process will be carried out by random sampling to the study groups. In the First Group (control group), patients will be followed in HF outpatients units according to the usual established protocol. In the Second Group (intervention group), patients will participate in the ACP program. Treatment for HF will be the same in both groups. The research team has developed the ACP program based on the published bibliography on ACP and advanced HF. The four hospitals mentioned will start recruiting at the beginning of the study. The principal investigator will be responsible for interrupting recruitment once the sample size has been reached (Additional file 1).

In the first visit, patients from both control and intervention groups will complete the following questionnaires with the help of researchers:

- PAM (Patient Activation Measure) Test (Table 2) [16]: test to measure the activation (participation and self-management) of the patient in decision-making. It evaluates the knowledge, skills and confidence of patients’ self-management classifying patients in levels of self-management activation. It also values the degree of patients’ implication in making decisions about their lives (in their self-management). This procedure has been previously applied to frail elderly patients in a Dutch study in order to measure patients’ activation in decision making. The test divides the patients in four levels according to their decision-making capacity:

**Table 2:** The PAM 13 measurement instrument

PAM assesses a consumer’s knowledge, skills and confidence for self-management. PAM segments people into one of four progressively higher levels of activation. Below are some statements that people sometimes make when they talk about their health. Please indicate how much you agree or disagree with each statement as it applies to you personally by circling your answer. Your answers should be what is true for you and not just what you think others want you to say		
1	When all is said and done, I am the person who is responsible for taking care of my health	Disagree strongly Disagree Agree Agree strongly N/A
2	Taking an active role in my own health care is the most important thing that affects my health	Disagree strongly Disagree Agree Agree strongly N/A
3	I am confident I can help prevent or reduce problems associated with my health	Disagree strongly Disagree Agree Agree strongly N/A
4	I know what each of my prescribed medications do	Disagree strongly Disagree Agree Agree strongly N/A
5	I am confident that I can tell whether I need to go to the doctor or whether I can take care of a health problem myself	Disagree strongly Disagree Agree Agree strongly N/A
6	I am confident that I can tell a doctor concerns I have even when he or she does not ask	Disagree strongly Disagree Agree Agree strongly N/A
7	I am confident that I can follow through on medical treatments I may need to do at home	Disagree strongly Disagree Agree Agree strongly N/A
8	I understand my health problems and what causes them	Disagree strongly Disagree Agree Agree strongly N/A
9	I know what treatments are available for my health problems	Disagree strongly Disagree Agree Agree strongly N/A
10	I have been able to maintain (keep up with) lifestyle changes, like eating right or exercising	Disagree strongly Disagree Agree Agree strongly N/A
11	I know how to prevent problems with my health	Disagree strongly Disagree Agree Agree strongly N/A
12	I am confident I can figure out solutions when new problems arise with my health	Disagree strongly Disagree Agree Agree strongly N/A
13	I am confident that I can maintain lifestyle changes, like eating right and exercising, even during times of stress	Disagree strongly Disagree Agree Agree strongly N/A

  - *Level 1* They do not feel responsible for their own health and care. Health management is overwhelming for them, considering all the problems of life. They lack confidence in their ability to manage health. They have few solving problem skills and poor coping skills. They may not be fully aware of their behaviour (Score 47.0 or lower).
  - *Level 2* They may lack basic understanding about their condition, treatment options, and/or self-care. They have little experience making decisions. They consider their doctor as the decision maker. They have little confidence in their ability to manage their health (47.1–55.1).
  - *Level 3* They know the basic facts of their illness and treatments. They have some experience making decisions and some confidence in the management of certain aspects of their health (55.2–67).
  - *Level 4* They have made most of the decisions, but they may have difficulties in maintaining behaviours over time or in stressful situations (67.1 or higher).
- MLWHFQ Questionnaire (Minnesota Living with Heart Failure Questionnaire) [17]: validated to analyze the quality of life in patients with HF (Spanish version). Scoring range: 0 to 105 points: The higher the score obtained, the poorer the quality of life of patients. Patients wonder to what extent their illness has prevented them from living the way they wanted last month. Questions refer to signs and symptoms of the disease, social relationships, physical, sexual activity, work and emotions. It is self-administered, with Likert response options, ranging from 0 (quality of life not affected) to 5 (maximum impact on quality of life). The global MLWHFQ score is obtained by adding the 21 items scores (range: 0–105); the highest value corresponds to the worst HRQL. It assesses the impact of chronic heart failure in two dimensions: the physical dimension based on eight items (range: 0–40) and the emotional dimension consisting of five items (range: 0–25).
- Zarit questionnaire about caregiver's burden [18]. This interview measures caregivers’ degree of subjective overload in relation to chronic patients. It consists of 22 items that collect caregivers’ feelings. Each feeling scores on a frequency gradient that ranges from 1 (never) to 5 (almost always). Interpretation:
  - or < 45 points: no overload
  - 47–55 points: slight overload
  - or > 55 points: intense overload

In addition, the following data will be collected: epidemiological data (date of birth, sex), presence of diabetes mellitus, dyslipidaemia, alcohol and tobacco consume, thyroid disease, etiology of cardiomyopathy, left ventricular ejection fraction, heart devices, Charlson index, hours of weekly physical exercise, sociocultural characteristics (marital status, housing, people living in the house, education, employment status, economic income per year, religion) and also characteristics of the main caregiver (number of caregivers and who is the main caregiver, relationship with the patient, caregiver's employment status).

After 1 year of follow-up, the questionnaires will be submitted again to all patients and three new questionnaires will be proposed:

- Patient's wishes checklist: Checklist to test the fulfilment of planned patient's wishes. Two parameters (patient participation in the decision-making thought PAM Test and fulfilment of their wishes) allow us to determine whether decision-making in advanced HF is actually being effective.
- Satisfaction questionnaire (Table 3): Created “ad hoc”, it aims to assess the level of satisfaction with the program. Its outcome will help improve the future implementation of the program.

**Table 3:** Satisfaction questionnaire

Answer YES or NO	YES	NO
Did you understand what the program was about?		
Do you feel you received enough information, orally or in writing, for your understanding?		
Did you feel that you could easily access the doctors responsible for the program to answer your questions?		
Do you think that the doctors responsible for the program have the necessary skills and knowledge?		
Did you understand without difficulty what these professionals communicated in each interview?		
Has the program met your expectations and needs?		
Did you find it easy to get to the office and/or day hospital?
- Dying and Death quality questionnaire (QODD) [19]: This questionnaire measures the quality of the dying process. It will be submitted to the relatives of patients who passed away during the year of follow-up.

The Satisfaction questionnaire has been developed for this study. The other questionnaires had already been previously published.

All data will be registered in a database created for the purpose of the study.

### Sample size calculation

The sample size estimate has been made assuming a power of 80%, a confidence level of 95% in order to detect a standardized significant difference of at least 0.5. We consider 0.5 to be a clinically relevant difference based on the article by Norman et al. [20], being 128, the minimum sample size needed to detect this difference, 64 people in each group. A 10% sample size is added to compensate possible losses or errors in the follow-up, which represents 140 patients.

### Randomization

Subjects’ allocation to the two groups will be carried out by simple random sampling stratified by hospital. This sampling will be processed by a computer program that randomizes the patients in each hospital. Randomization will result in two lists of random numbers between 1 and 50 in each hospital. In each research center there will be 50 opaque and sealed envelopes numbered from 1 to 50. Each envelope will contain the group for that particular patient (intervention vs. control). The number on each envelope will correspond with the entry order number of each subject and will be opened once the patient has been included in the study. Each hospital must recruit a minimum of 20 patients and a maximum of 50 patients. Recruitment will end when sample size is reached.

### Statistic analysis

An intention-to-treat analysis will be carried out. In the descriptive analysis, categorical variables will be presented with their frequency distribution and 95% CI and quantitative variables using mean (SD) when the distribution is normal and in case of non-normality, the median (RIQ).

Qualitative variables will be compared using the Chi2 test or Fisher's exact test, in the event that more than 25% of the expected values are less than 5. For quantitative variables of normal distribution, will be employed Student's t-test or the Mann–Whitney *U* non-parametric tests. In case of non-normal distributions analysis of variance (ANOVA) will be carried out. To compare quantitative variables in more than two groups, the non-parametric Kruskal–Wallis test will be used. In all cases the distribution of the variable will be checked against the theoretical models and the hypothesis of variance homogeneity will be contrasted. For quantitative outcome variables, multiple linear regression analyses will be carried out. The analyses will be carried out with the statistical package SPSS 17.0, and the level of significance will be set at 5%.

### Funding and Trial registration

The study is supported by a public grant from the *Fondo de Investigaciones Sanitarias* (Instituto de Salud Carlos III); FIS grant number: PI19/01647.

ClinicalTrials.gov Identifier: NCT04424680. Date of registration: June 9, 2020. https://clinicaltrials.gov/ct2/show/NCT04424680?term=NCT04424680&draw=2&rank=1.

### Ethical aspects

The present study has been approved by the Clinical Research Ethics Committee (CREC) of the Fundación Alcorcón Hospital and it will also be approved by the CREC of each research center. Both the staff involved in the project and researchers know and vow to respect local and international regulations for human experimentation, including the Helsinki declaration and its revisions, the Belmont report and other related documents.

Data confidentiality will respect the Law on Data Protection (Organic Law 5/92 of October 29 on the regulation of the automated processing of personal data, BOE October 30, 1992 modified by the Organic Law 15/1999, of December 13, Protection of Personal Data and Law 41/2002, of November 14).

Patients will be informed in detail about the protocol and will sign a written informed consent before being included in the study.

### Limitations of the study

Even if the ACP program intervention is homogeneous in both groups, and everyday clinical practice is very similar in all centers, the organization of each HF Unit can vary. Despite this fact, this scenario parallels real clinical variability. Even though clinical management will be similar in both groups, as an open clinical trial there is a risk of expectation and attention bias. This might make it difficult to ensure that researchers' higher engagement with patients in the intervention group will not skew results. Finally, the study is restricted to patients with advanced HF, and therefore its conclusions and extrapolation to other advanced chronic pathologies is limited.

## Discussion

An ACP program involves planning decisions for patients who are at risk of losing the ability to decide. To do this, patients have to be adequately informed and supported by the healthcare team, so that they can establish their preferences regarding health. Possible future clinical scenarios must be foreseen so that the patients can express their wishes.

All these possible scenarios and the patient’s values must be known by their healthcare team and entered into their medical record, so as to ensure the patients’ wishes are respected if there is a situation in which they are unable to express their care preferences.

The characteristics of patients with advanced HF make them a model for designing ACP programs, given the high prevalence of this disease, the progressive increase in its incidence and it’s clinical characteristics: a low life expectancy in advanced phases and the ability to foresee future clinical scenarios on which is possible to plan decision-making [21]. Until now, the efficacy of this type of program has not been measured, so this Clinical Trial can provide relevant data for future ACP projects.

## Supplementary information

**Additional file 1.** Flow Diagram.

## Data Availability

Not applicable (no data or study results yet available).

## Abbreviations

ACP: Advanced Care Planning
HF: Heart failure.
AD: Advance directives
ACCF/AHA: American College of Cardiology Foundation/American Heart Association
PAM: Patient activation measure
SD: Standard deviation
