# Supplementary material for: Evaluating the efficacy of an Advanced Care Planning Program for Health Decisions in patients with advanced heart failure: protocol for a Randomized Clinical Trial
Source: BMC Cardiovasc Disord. 2020 Oct 21;20:456. doi: 10.1186/s12872-020-01738-0 (PMC7579909; doi:10.1186/s12872-020-01738-0)
Supplement: Supplementary file 1 — Additional file 1. Flow Diagram. [file 12872_2020_1738_MOESM1_ESM.docx]

**Flow Diagram**

Lost to follow-up (approximately) (n= 1)

Discontinued intervention (approximately) (n=5); main cause: death.

Lost to follow-up (approximately) (n= 1)

Discontinued intervention (approximately) (n=5); main cause: death.

## Follow-Up

Analysed (n= 64)

## Analysis

Analysed (n= 64)

## Enrollment

Will not receive allocated intervention (control group) (n= 70)

## Allocation

Allocated to intervention (n= 70)

Randomized (n = 140)

Excluded approximately (n = 20)

♦  Not meeting inclusion criteria or,

♦  Declined to participate or,

♦  Other reasons.

Assessed for eligibility (n= 160)
